# AutoCLEM: An Automated Workflow for Correlative Live-Cell Fluorescence Microscopy and Cryo-Electron Tomography

**DOI:** 10.1038/s41598-019-55766-8

**Published:** 2019-12-16

**Authors:** Xiaofeng Fu, Jiying Ning, Zhou Zhong, Zandrea Ambrose, Simon Charles Watkins, Peijun Zhang

**Affiliations:** 1 Department of Structural Biology, University of Pittsburgh School of Medicine, Pittsburgh, PA 15260 USA; 2 Department of Microbiology and Molecular Genetics, University of Pittsburgh School of Medicine, Pittsburgh, PA 15260 USA; 3 Department of Cell Biology and Physiology, University of Pittsburgh School of Medicine, Pittsburgh, PA 15260 USA; 4 Division of Structural Biology, Wellcome Trust Centre for Human Genetics, University of Oxford, Oxford, OX3 7BN UK; 5 Electron Bio-Imaging Centre, Diamond Light Sources, Harwell Science and Innovation Campus, Didcot, OX11 0DE UK

**Keywords:** Biophysics, Biological fluorescence

## Abstract

Correlative light and electron microscopy (CLEM) combines the strengths of both light and electron imaging modalities and enables linking of biological spatiotemporal information from live-cell fluorescence light microscopy (fLM) to high-resolution cellular ultra-structures from cryo-electron microscopy and tomography (cryoEM/ET). This has been previously achieved by using fLM signals to localize the regions of interest under cryogenic conditions. The correlation process, however, is often tedious and time-consuming with low throughput and limited accuracy, because multiple correlation steps at different length scales are largely carried out manually. Here, we present an experimental workflow, AutoCLEM, which overcomes the existing limitations and improves the performance and throughput of CLEM methods, and associated software. The AutoCLEM system encompasses a high-speed confocal live-cell imaging module to acquire an automated fLM grid atlas that is linked to the cryoEM grid atlas, followed by cryofLM imaging after freezing. The fLM coordinates of the targeted areas are automatically converted to cryoEM/ET and refined using fluorescent fiducial beads. This AutoCLEM workflow significantly accelerates the correlation efficiency between live-cell fluorescence imaging and cryoEM/ET structural analysis, as demonstrated by visualizing human immunodeficiency virus type 1 (HIV-1) interacting with host cells.

## Introduction

Cryogenic correlative light and electron microscopy (cryoCLEM) has become a valuable tool for investigating the high-resolution ultra-structures of specific biological entities and events *in situ* in their near-native states, owing to the combined advantages of both imaging modalities. On one hand, fluorescence light microscopy (fLM) enables spatial localization and analyses of dynamic properties of molecules in cellular processes. Particularly, proteins, macromolecular complexes and organelles that have been labeled with fluorescence probes can be localized, tracked, and distinguished from the crowded content of the cell, however, with limited spatial resolution (dozens of nanometers at best in super-resolution mode^1^). On the other hand, cryo-electron tomography (cryoET) has emerged as a powerful technique for visualizing three-dimensional (3D) cellular structures at high resolution in their near-native state and natural environment^2^. With the recent development of subtomogram averaging techniques, cryoET is now able to generate detailed views of cellular components at sub-nanometer resolutions^3–6^. However, the information obtained from cryoET is limited to 3D snapshots of a biological event at specific time points. To achieve a time-lapse panorama and capture meaningful scenes during biological processes, such as virus entry, budding or cell signaling, combined live-cell imaging and high-resolution cryoET with subtomogram averaging using cryoCLEM, is especially desirable^7,8^.

One challenging aspect of cryoCLEM is to image fluorescence signals at cryogenic temperature in order to keep the sample vitrified. For this purpose, several groups have developed cryo-light microscopy stages^7,9–14^, cryofLM probes^15,16^ and methods^17–21^ for cryofLM imaging. Recently cryofLM devices have become available from several commercial sources (FEI Corrsight, Leica EM CryoCLEM^22^, Zeiss CryoAiryscan and Linkam). Such systems have been successfully applied to image neurons, viruses, mitochondria and many other cellular components^8,12,14,19,23–28^. To improve the accuracy and efficiency of cryoCLEM, various probes (visible in both fLM and EM) have been employed to guide the correlation step; these include toner particles^29^, FluoroNanogold (FNG)^30^, quantum dots (QDs)^31^, 3,3′-diaminobenzidine (DAB after photoconversion)^32^, fluorescent dye beads^13,21,22,33^, and cathodoluminescence pointers^34^. Further, on the EM side, automation software, such as Leginon^35^, serialEM^36^ and ThermoFisher/FEI EPU/TOMO, have allowed users to generate grid atlas maps and to import fLM maps for alignment and registration.

However, the entire process of cryoCLEM is (i) tedious and time-consuming due to difficulties in localizing the corresponding region of interest (ROI) under cryoEM^19,20^, (ii) inefficient with low throughput due to multiple steps of manual operation and correlation, and (iii) difficult due to low signals in cryoEM that make direct correlation a challenge. To simplify the cryoCLEM procedure and to make it more robust and accurate, we developed AutoCLEM, a workflow employing fluorescent fiducial beads for automated alignment of correlative images in three sequential steps, from low-resolution fLM to low and medium magnification cryoEM, and to high resolution cryoET. We applied it to examine the early stages of infection by human immunodeficiency virus type 1 (HIV-1) and demonstrate a greatly reduced investment of time for correlation processes (10 fold) as well as improved accuracy (5 fold).

## Results

### Overview of autoCLEM workflow

**Figure 1:**
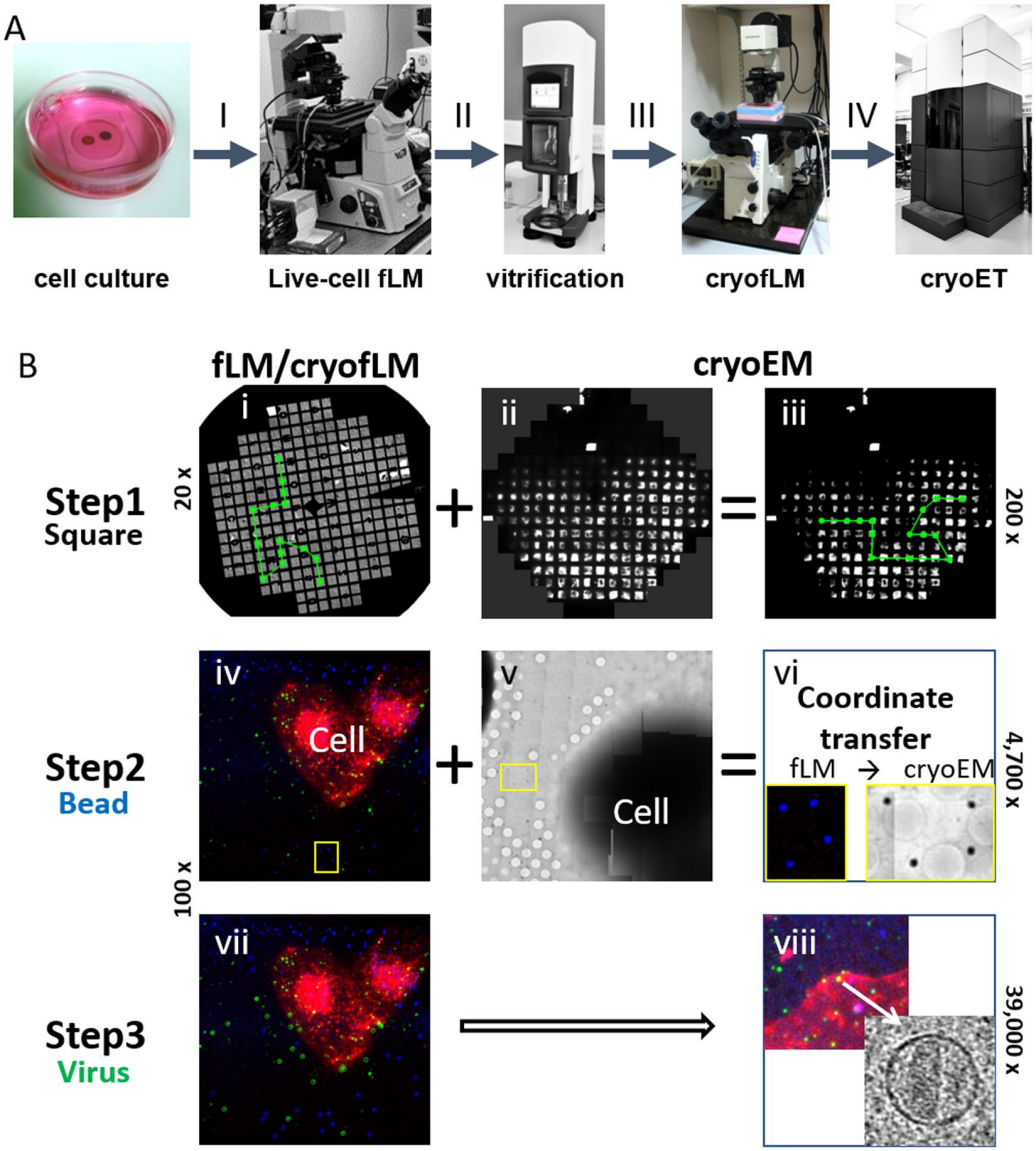
Workflow for AutoCLEM. (**A**) Four main steps in cryo-correlative light and electron microscopy (cryoCLEM). (**B**) Schematic of AutoCLEM. Three sequential steps of automated correlation are illustrated. Step1: Low magnification fLM (i, 20×) and cryoEM (ii-iii, 200×) for grid overview, showing that 11 target squares (green dots) are automatically correlated. Step 2: High magnification fLM image (100×) to identify areas of interest (iv, yellow box, four blue beads) and the corresponding cryoEM image recorded at a medium magnification (4,700×) (v-vii, four beads). Based on beads, a coordinate transfer matrix is generated between fLM (iv) and cryoEM (v). Step 3: High magnification (39,000×) for high resolution cryoET. Particles of interest (vii, green) are localized for cryoET data collection (viii) by applying the transform obtained from step 2. Beads, HIV-1 particles and cells are in blue, green and red, respectively.

The conventional cryoCLEM experiment usually involves four main steps: I) imaging fixed or live cells with fLM, II) plunge-freezing of cells on EM grids, III) cryofLM imaging, and IV) cryoEM/ET data collection (Fig. 1A). The key to successful cryoCLEM is a reliable and effective correlation between fLM in step III and cryoEM/ET in step IV. As illustrated in Fig. 1B, the AutoCLEM workflow comprises three levels of correlation between fLM/cryofLM and cryoEM/ET, to automatically localize targets of interest (e.g. HIV-1 virus particles) for cryoEM/ET structural analysis. The experimental setup for cell culture on EM grids and subsequent viral infection is shown in Supplementary Fig. S1. The 200 nm FluoSphere beads were pre-coated on the backside of the EM grids to avoid their endocytosis by cells (Supplementary Movie S2, Supplementary Fig. S3) and to minimize bead movement or loss during plunge freezing.

The first step of correlation is at the level of grid-square, indicated as ‘square’ correlation (Fig. 1Bi-iii). It is not trivial to directly align these two overview maps from fLM (Fig. 1Bi) and cryoEM (Fig. 1Bii,iii), because the cryoEM map clearly has different intensity characteristics from fLM map, including the presence of intensity gradients with totally dark regions due to the thick ice. Nevertheless, the common features such as the grid bars and characters on the ‘finder’ grid aid alignment of the two maps. After rescaling and thresholding the intensity of the maps, the two overview maps can be aligned automatically using a homemade script in AutoCLEM software (Supplementary material) by cross-correlation. This alignment is done with the IMOD command ‘xfalign’ where the best match is found at the highest cross-correlation value by varying rotation angle and slightly refining magnification. The coordinates for the center of interested grid-squares from fLM are mapped onto the corresponding squares in cryoEM (Fig. 1Bi,iii, green dots). This ‘square’ correlation can tolerate certain grid defects or deformations, which are common to cryoEM grids since they are easily damaged during freezing, loading or transfer. In fact, using a cryoCLEM sample stage that accepts an autogrid^22^ or a polara cartridge^7^ minimizes direct manual handling of EM grids, thus, greatly reduces the artifact.

The next step of correlation is at the fluorescent bead level, indicated as ‘bead’ correlation (Fig. 1Biv–vi). This step generates a transformation matrix between the fLM image at 100 × magnification (Fig. 1Biv) and the cryoEM grid-square map at 4,700 × magnification (Fig. 1Bv) using 200 nm fluorescent beads. The grid-square map (Fig. 1Bv) is a 12 × 12 montage of cryoEM images recorded at 4,700×, a magnification at which the beads are visible and easily distinguished from ice or other contaminants (Fig. 1Bv-vi). Typically, at least 4–5 beads close to the cell periphery are manually or automatically picked from the fLM image (Fig. 1Biv) and from the cryoEM grid-square map (Fig. 1Bv). A Matlab script (Supplementary material) is developed to compute the transformation matrix (Fig. 1Bvi), which allows fitting of parameters including magnification, rotation, translation, and distortion between these two maps based on the selected beads.

The last step of correlation, indicated as “virus” correlation in Fig. 1, is to convert the fLM-extracted ROI coordinates (in this case, virus particles) into cryoEM stage positions by applying the transformation matrix generated from step 2. A high magnification cryoET tilt series can then be collected automatically at the computed position for the ROI (Fig. 1Bviii), considering a calibrated microscope alignment between medium (4,700×) and high (39,000×) magnifications. These three successive steps are entirely automatic in most cases, with the exception when fLM signals are complicated or overcrowded calling for the need to manually pick viruses and beads. We describe detailed autoCLEM workflow and results in the following sessions using HIV-1 infection as an example.

### Automated fLM grid-scan for target selection (Step 1)

**Figure 2:**
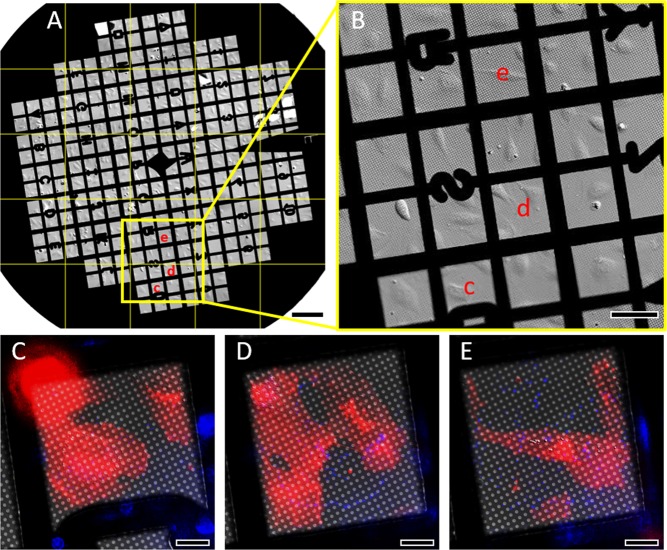
Automatic grid scan with fLM and target square selection. (**A)** An EM grid was automatically scanned for a 5 × 5 array with fLM at 20x DIC mode, stitched for grid montage. (**B**) An area (yellow box in A) is zoomed in to clearly view cell concentration, morphology and distribution in each grid square, to allow pre-selection of appropriate squares. These pre-selected squares (‘c-e’ in B) are then further inspected by automatic collection in three channels (DIC, cells (red) and beads (blue)) with fLM at 100x (**C**–**E**). The cell membrane dye (red) shows much clearer cell boundary than DIC channel (C&D) because the HUVEC cell periphery is very thin. The blue signal from beads help to guide selection of regions with proper bead distribution. The scale bar is 200 µm in A, 100 µm in B, and 20 µm in (**C**–**E**), separately.

In previously reported CLEM methods, the correlation process required manually recording the grid-squares selected for confocal fLM imaging and later finding them in cryoEM. This could be difficult when 1) no recognizable or usable feature on the ‘finder’ EM grid is present to help correlate grid-squares; or 2) the grid is flipped during loading for cryoEM. To get around this manual step, we utilized a new feature of the fLM software NIS-Elements, ‘scan large map’, to automatically generate an overview at 20 × magnification, Differential Interference Contrast^37^ (DIC) map (5 × 5 tiles are shown) of the entire EM grid in less than 5 minutes with auto-focusing (Fig. 2A). Furthermore, the live-cell fLM overview map provides high quality images that allow for examination of cells and selection of regions for high magnification (100×) imaging. For example, the user can simply use this map to select the most appropriate grid-squares or regions over the entire grid within which only 1–2 cells are located (too many crowded cells will produce too thick ice during plunge freezing), and where the cells are mostly spread out, between two mitosis phases. This fLM overview map is correlated automatically to the cryoEM grid scan overview map (Supplementary Fig. S2) to facilitate selection of targeted grid-squares for the next level of correlation for higher precision.

The selected grid-squares, such as those indicated in Fig. 1B (green dots) and Fig. 2A,B (labeled as c-e), are then further inspected at higher magnification (100×) with automatic recording of DIC and several fluorescence channels, including cell tracker (red) and fluorescent fiducial beads (blue). The cell tracker signal is particularly useful for determining the cell boundary since, for cryoEM, we are interested in the very thin cell periphery, which is almost invisible in the DIC mode, but clearly revealed with cell tracker (Fig. 2C–E). Meanwhile, the fluorescent bead distribution is also clearly exhibited (Fig. 2C–E, blue). Criteria for selecting good grid-squares include 1) the presence of only 1~2 cells with the cell periphery located close to the center of the square, and 2) the presence of at least 4~5 beads around the cell periphery of interest.

### Automated confocal live-cell microscopy (Step 2)

**Figure 3:**
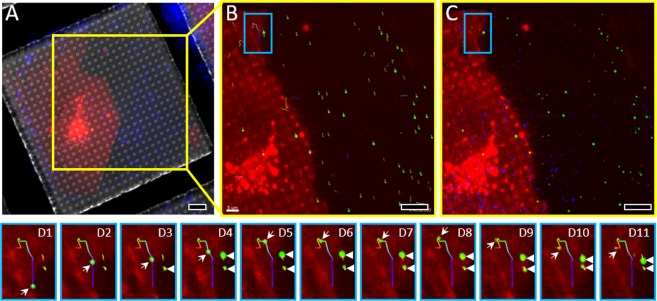
Automated confocal live-cell microscopy. (**A**) A typical grid square with overlaid signals from three channels, DIC, red (cells) and blue (beads). (**B,C**) The last time point of confocal image (yellow box area in panel A, shown overlaid signals from red (cells), green (HIV-1 viruses) (**B**) and blue (beads) (**C**). The green viruses are tracked in 3D from time-lapse confocal live-cell images, shown in B (thin line tracks). (D1-11) Enlarged views of a time-lapse series of an infecting virus (in cyan box area in **B,C**) from time 0 to 40 minutes with an interval of 4 minutes. The scale bars are 10 µm in (**A**–**C**).

Live-cell imaging with fLM is a key step in capturing cellular processes at a stage of interest, for example, during the early stages of HIV-1 infection. In this case, high-speed confocal live-cell imaging at pre-selected grid squares was performed during the first 40–60 minutes after the addition of HIV-1 virus particles (Fig. 3, green). These virus particles carry a mutation E45A on the capsid protein (CA), which causes the capsid to be hyper-stable^38,39^ making it easier to capture intact capsids in imaging studies compared to wild-type virus particles^7^. To image HIV-1 and host cell interactions by cryoET, we targeted thin (<300 nm) periphery regions of the cell. High-speed time-lapse confocal fLM images were automatically recorded, with Z-sections from the fiducial beads located on the back-side carbon film to the top surface of the cell (Fig. 3, Supplementary Movie S1). Both virus particles (green) and cells (red) were imaged simultaneously during the entire time-lapse confocal live-cell imaging period, while the image of beads (blue) was collected only once, at the end of confocal live-cell imaging (Fig. 3C). From the live-cell confocal series, we were able to track HIV-1 particles in 3D (Supplementary Movie S1), revealing fast virus particle movements over the 40 minute post-infection when particles were associated (overlapped) with the cell (Fig. 3B); however, a majority of virus particles were relatively stationary and located away from the cell (Fig. 3B). Focusing on the area marked with a cyan box in Fig. 3B, one virus particle displayed rapid movement in a curvilinear path in the cell periphery (Fig. 3D1–11, arrows), consistent with previous observations that particles traffic along microtubules within the cytoplasm^8,40^. Within the same area, two other virus particles displayed much less motion (Fig. 3D1–11, arrowheads).

### Automated fiducial correlation and virus particle localization (Step 2–3)

**Figure 4:**
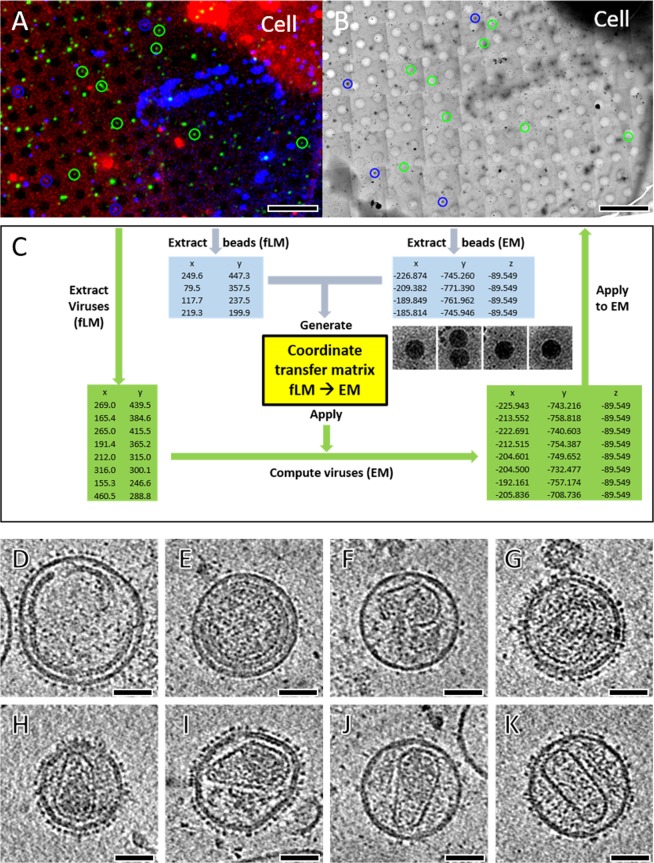
Automated (or semi-automated) correlation of beads and localization of HIV-1 particles. (**A**) The last confocal fLM image for a selected square with red (cells), green (viruses) and blue (beads) signals. (**B**) The corresponding cryoEM image at medium magnification (4,700×) showing bead positions. Four individual beads or bead aggregates (blue circles) were selected for correlation. (**C**) The illustration of workflow for bead correlation and viral localization. The coordinates of four beads (in blue) both from fLM and cryoEM images are extracted, from which the coordinate transfer matrix (in yellow) is generated with the consideration of scaling, rotation, translation and distortion. Applying the transfer matrix to virus coordinates (in green), extracted from fLM image, enables computing their coordinates (in green) in cryoEM. (**D**–**K**) Central slices of reconstructed tomograms from 8 correlated virus particles in panel (**A**,**B**) display various morphologies: defective immature particle (**D**), immature particle (**E**), defective mature particle (**F**), maturation intermediates (**G**–**I**) and mature particles (**J**,**K**). The scale bars are 10 µm in A-B and 50 nm in (**D**–**K)**, respectively.

Confocal live-cell images of virus particles (green), fluorescent beads (blue) and cells (red) were recorded simultaneously at the end of time-lapse series, and these were projected in 2D, as visualized in the overlay image (Fig. 4A). The information in the Z direction was omitted in this case but could be very useful when cryoCLEM processing includes cryo-focused ion beam (cryoFIB) milling in a scanning electron microscope. From the 2D images, four blue beads were manually selected using IMOD software^41^ (Fig. 4C, blue box). Once the corresponding cryoEM grid-square map was acquired (Fig. 4B), the same beads identified in the fLM projection image (Fig. 4A) were identified in the cryoEM map, and their corresponding coordinates automatically saved in a SerialEM navigation file (Fig. 4C, blue box). A Matlab script was developed (Supplementary material) to calculate the transformation matrix between fLM and cryoEM map based on the bead coordinates from the two microscope systems. The core part of this script is called ‘estimateGeometricTransfrom’. It can calculate the scaling, translation, rotation and distortion between fLM and cryoEM images. The script works quite well even when there appears to be some grid bending, which frequently happens during freezing or grid handling. The initial coarse positioning error, measured at a low cryoEM magnification (4,700×), is about 0.74 µm (n = 33), which is further reduced to 0.13 µm (n = 17), measured at high magnification for cryoET (39,000×).

After generating the coordinate transfer matrix (Fig. 4C, yellow box), we selected eight extracellular virus particles, as these were expected to have been stationary before freezing (Fig. 4A, green circles). The virus coordinates were extracted from the fLM 2D projected image and converted to cryoEM stage positions (Fig. 4C, green boxes). The SerialEM navigator file was then updated with the calculated virus coordinates in EM, followed by automated tomography data acquisition through all selected virus particles with SerialEM. The error for positioning virus particles was analyzed to be ~100 nm (n = 13), which is well within the tolerant range for cryoEM imaging at the high magnification, typically with an imaging frame of ~1.0 µm × 1.0 µm for a pixel size of 2.8 Å. The tomographic slices from the eight selected virus particles (Fig. 4A, green circles) are presented in Fig. 4D–K. These virus particles display a variety of morphologies, including defective immature particles (D), immature particles (E), defective mature particles (F), maturation intermediates (G-I) and mature particles with clear conical-shaped cores (J-K)^42^. All viruses show clear envelope spikes as they are located outside of cells.

### Auto-cryoCLEM of HIV-1 infection (Step 3)

**Figure 5:**
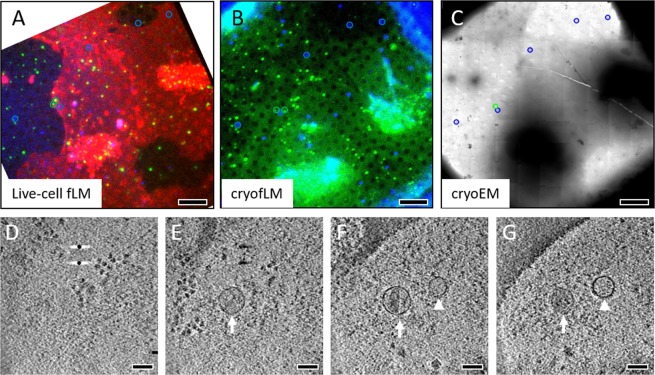
Tomographic imaging of HIV-1 particles bound to a cell using cryo-AutoCLEM. (A-C) The grid square images are displayed for confocal fLM at the last time point (**A**), cryofLM after freezing (**B**) and the cryoEM medium magnification scan (**C**). The beads were pre-coated on the EM grid and show a fixed pattern (blue circles). The virus particles (green circles) moved during a time gap of few minutes, between live-cell fLM and plunge freezing. The virus coordinate in cryoEM (**C**) was computed from the coordinate extracted from cryofLM image (**B**) using the transfer matrix generated from the bead coordinates (blue circles). (**D**–**G**) Four successive slice views of the tomogram acquired from the area containing correlated virus particle (arrow), along with an exosome (arrowhead). The scale bar is 10 µm in (**A**–**C**), and 100 nm in (**D**–**G**), respectively.

The initial AutoCLEM work was performed with confocal live-cell fLM at room temperature. This works especially well for static objects that do not move significantly during the 5–10 minutes gap between the last fLM image and plunge-freezing. However, in cases when the objects of interest are dynamic, for example the infecting virus particle shown in Fig. 3D, a direct correlation between cryofLM and cryoEM images after plunge-freezing is necessary. We tested AutoCLEM performance using the cryofLM setup that we reported previously^7^, by first collecting fLM images at the end of the confocal live-cell series and then cryofLM images after plunge-freezing. As shown in Fig. 5, both 2D projected confocal live-cell fLM (Fig. 5A) and cryofLM (Fig. 5B) images, before and after freezing respectively, displayed a similar pattern for the fluorescent beads, as expected (blue circles). Using the AutoCLEM script, the location of a virus particle identified in the cryofLM image was mapped onto the cryoEM image for tomography data collection (Fig. 5C, green). A mature HIV-1 virus bound to the cell surface and prior to fusion was captured and imaged. The slice views of the corresponding tomogram show ribosome molecules inside the cell (Fig. 5D,E), followed by the enveloped virus particles bound to the cell surface below (Fig. 5E–G, arrow), alongside an exosome (Fig. 5F,G, arrowhead).

## Discussion

The EM grids used in this protocol are Au or Ni finder Quantifoil grids, illustrated in Supplementary Fig. S1. The finder landmarks can be easily identified in both fLM and low magnification cryoEM and are necessary for three reasons: 1) the landmarks help users to identify the sample side before plunge freezing; 2) the landmarks help to identify whether the grid is flipped during cryoEM loading; 3) the landmarks help manual grid-square correlation in cases when the ice on the frozen grid is too thick. The use of nickel grids aids autofocus functionality in light microscopy during grid scan, but with a caveat of inducing image/beam shift during tomography data collection.

We have tested different FluoSphere dye beads for their utility as fiducial markers. The 100 nm bead is bright enough to be visible at 100× live-cell confocal fLM imaging and at 40× cryofLM but are difficult to distinguish at medium magnification cryoEM, especially around the cell periphery where the ice is relatively thick (~300 nm). The 1 µm bead also was not suitable, as beads of this size are easily blotted away during plunge freezing, whereas 100 nm and 200 nm sized beads remain on the EM grid. In addition, precisely determining the positions of the 1 µm beads in cryoEM is a challenge, as they often retain a significant amount of liquid during freezing, which obscures the shape of beads in cryoEM. We found that 200 nm beads work best as fiducial markers compared to either smaller or larger beads. We have also discovered that the beads must be pre-coated on the back side (non-sample side) of the EM grid, or added instantly before plunge freezing. If added to the sample side, the beads are taken up by the cells through endocytosis, as shown in Supplementary Fig. S3 and Supplementary Movie S2.

Previously reported CLEM methods include multiple steps of correlation, beginning from low magnification and employing either internal grid features (grid bar and edge or characters) or external particles (toner particles, random contamination) for manual correlation of grid-squares or areas of interest, followed by medium or high magnification correlation using immunogold, Quantum Dots, dye beads, holes on Quantifoil grid carbon, or even intrinsic cellular features as fiducial markers^13,21,22,29–34^. Since most of the correlations were performed manually, the procedures were very time-consuming and tedious. The autoCLEM workflow presented here comprises a near-fully automatic three-step correlation procedure; only the picking of fiducial beads in fLM image and cryoEM grid-square maps for calculating the transformation matrix is manual, and this takes less than 5 minutes. In principle, this manual aspect of the procedure can also be automated in AutoCLEM, but in practice, the variability of fluorescence signals among beads, the amount of ice contamination and variable ice thickness make this challenging. All together the grid-square correlation process (step 1) can be shortened to 10 minutes from 1 hour typically required in a manual effort, and the bead correlation and viral localization steps (steps 2–3) can be shortened to 10 minutes per square from previous 2 hours per square (Supplementary Table S1). Most importantly, the accuracy of target positioning is five times better (~500 nm to ~100 nm), which could be further improved when super-resolution cryoCLEM is performed^15^. The current challenge for a completely automated process is the frequent mismatch of bead patterns between fLM/cryofLM and cryoEM, due to missing fiducial beads in either system. We are developing methods to improve the detection reliability and correlation accuracy towards a fully automated AutoCLEM, as demonstrated in Supplementary Fig. S4, in which a correlation of beads was established even though the detection of beads was incomplete in both systems. This resulted in automatic localization of virus particles (Fig. S4F–G, pink circles) in cryo grid-square map.

## Methods

### Production of VSV-G pseudo-typed HIV-1 particles

As previously described^7^ E45A HIV-1 was produced by transfecting 293T cells with three plasmids: pL-VSV-G (a kind gift from M. Emerman), peGFPC3-Vpr (a kind gift from T. Hope), and a proviral plasmid, pNLdE-luc, encoding HIV-1_NL4-3_ with deletions in *env* and with the luciferase gene in place of *nef*^43^. Transfections were performed in 293T cells with lipofectamine 2000 (Invitrogen, Carlsbad, CA) in the cell medium DMEM (10% FBS Pen/Strep/Glu) (Gibco). Virus infectivity was determined on GHOST-R3/X4/R5 cells and capsid levels were measured by p24 ELISA (Zeptometrix, Buffalo, NY).

### Cell culture on cryoEM grids

Au or Ni R2/2 Quantifoil ‘finder’ EM grids (Quantifoil Micro Tools GmbH, Jena, Germany) were glow-discharged on the back-side of the grid at 20 mA for 20 seconds, coated with 1 µl 1000x diluted FluoSphere 200 nm blue beads (Invitrogen, Eugene, OR), and left to dry. The grids were then disinfected under UV light for 2 hours and treated with 50 µg/ml fibronectin (Sigma-Aldrich, from bovine plasma) on the front-side. Human umbilical vein endothelial cells (HUVEC) were cultured at 37 °C with 5% CO_2_ in EGM™-2 BulletKit™. Cultures at approximately 70–85% confluence were rinsed with 5 ml room temperature HEPES Buffered Saline (HEPES-BSS) and incubated in Trypsin/EDTA solution (0.5 ml Trypsin/EDTA for 60 mm culture dishes). After about 90% of the cells were rounded, the cells were diluted in cell medium, to a density of 0.8–1 × 10^5^ cells/ml, and plated (2 ml/dish) onto disinfected grids placed in glass-bottom culture dishes (MatTek Corporation, Ashland, MA) with the cell side up. Each dish was incubated overnight.

### Live-cell fluorescence imaging

The HUVEC cells were stained for 10 minutes by addition of 1.0 µl of cell tracker, CellMask Deep-Red (ThermoFisher, Waltham, MA, USA.) to the culture medium. After rinsing and replacement of the culture medium, the cells were infected and imaged as follows. During these processes, careful attention should be paid to not disturb the EM grids in the dishes. For fluorescence live-cell imaging, a Nikon Ti widefield microscope, Bruker Optera confocal scan head and Andor Ultra EM CCD camera was used. A Tokai hit environmental chamber was used to maintain temperature and normal 5% CO_2_ tissue culture conditions. The system was controlled by NIS Elements software (Nikon instruments, Melville, New York). The EM grid was imaged in glass-bottom dish, which was centered and automatically scanned in DIC channel at 20 × magnification (20 × 0.75 NA plan apo lens), in 5 × 5 stitched tiles, covering the entire 3.05 mm EM grid (Fig. 2A). Grid-squares of interest were selected, and images of these regions were automatically acquired at 100 × magnification (100 × 1.49 TIRF) in DIC and two fluorescence channels (640 nm red for cell and 405 nm blue for bead). After the addition of 200 µl pseudo-typed E45A HIV-1, time-lapse confocal live-cell imaging was performed immediately on selected areas at 100 × using the high speed confocal head and EMCCD camera. Confocal images were recorded automatically every 5 minutes for 40 minutes, for two fluorescence channels (488 nm for virus and 640 nm for cell) with triggered sequence illumination and over an 8 µm Z space at 1.0 µm spacing. After the time-lapse confocal imaging was finished, a final set of confocal images were collected with an additional fluorescence channel at 405 nm (for fluorescent beads), along with the two previous fluorescence channels (488 nm for virus and 640 nm for cell). 3D reconstruction and virus particle tracking were performed using Imaris (Bitplane, Zurich, Switzerland).

### Cryo-electron microscopy and tomography

Immediately after fluorescence confocal imaging, 4 µl of 15 nm gold beads were applied to the EM grid, which was subsequently blotted and plunge frozen using an FEI Vitrobot (FEI, Hillsboro, OR) at ~100% humidity. The frozen grids were clipped into a Polara cartridge and imaged with a home-built cryo-sample stage to record cryo-fluorescence images, as described previously^7^.

The frozen grids were loaded to Polara G3 microscope (FEI Corp., OR.), equipped with a Gatan US4000 4k × 4k Ultrascan CCD (Gatan, Inc., Warrendale, PA) and a Falcon II direct detector (FEI Corp., OR.) camera, for cryoEM analysis and cryoET data collection. A cryoEM overview map (atlas, a low magnification montage, LMM) of the entire grid was recorded using serialEM, with which a 14 × 14 tiles LMM overview was recorded at 200 × on the Ultrascan camera. This LMM overview is correlated to the fLM tile images to find the location of the grid-squares of interest.

After the grid-squares of interest were located, a medium magnification montage (MMM) was recorded for each square at 4,700× on Falcon II camera with 20% overlap and 300 µm defocus. Using the correlative script developed in this study, coordinates of virus particles were determined, and tilt series were collected bidirectionally, with a tilt range of −60° to 60° and 3° interval, at a nominal magnification of 39,000×, defocus values between 5–8 µm and a total dose of ~100 e/Å^2^ on the Falcon II camera. The tilt series were aligned using 15 nm gold fiducial beads and reconstructed with weighted back-projection algorithm using the IMOD package^41^. The tomograms of viruses were denoised using 3D nonlinear anisotropic diffusion edge enhancing program implemented in IMOD with 6 iterations and a κ value of 0.8 to enhance visualization.

## Supplementary information

Supplementary Information

Movie 1

Movie 2

## Data Availability

The datasets generated during and/or analyzed during the current study are available from the corresponding author on reasonable request. The AutoCLEM Script is freely available from https://github.com/whatanamelikethis/ok/blob/master/CLEM_align.
