# Supplementary material for: AutoCLEM: An Automated Workflow for Correlative Live-Cell Fluorescence Microscopy and Cryo-Electron Tomography
Source: Sci Rep. 2019 Dec 16;9:19207. doi: 10.1038/s41598-019-55766-8 (PMC6915765; doi:10.1038/s41598-019-55766-8)
Supplement: Supplementary file 1 — Supplementary Information [file 41598_2019_55766_MOESM1_ESM.pdf]

Supplementary Information

for

AutoCLEM: An Automated Workflow for Correlative Live-Cell Fluorescence Microscopy and Cryo-Electron Tomography

Xiaofeng Fu, Jiying Ning, Zhou Zhong, Zandrea Ambrose, Simon Charles Watkins and Peijun Zhang*

This document includes

Supplementary figures1-4

Supplementary Tables 1

Supplementary Movies 1-2

Supplementary script 1

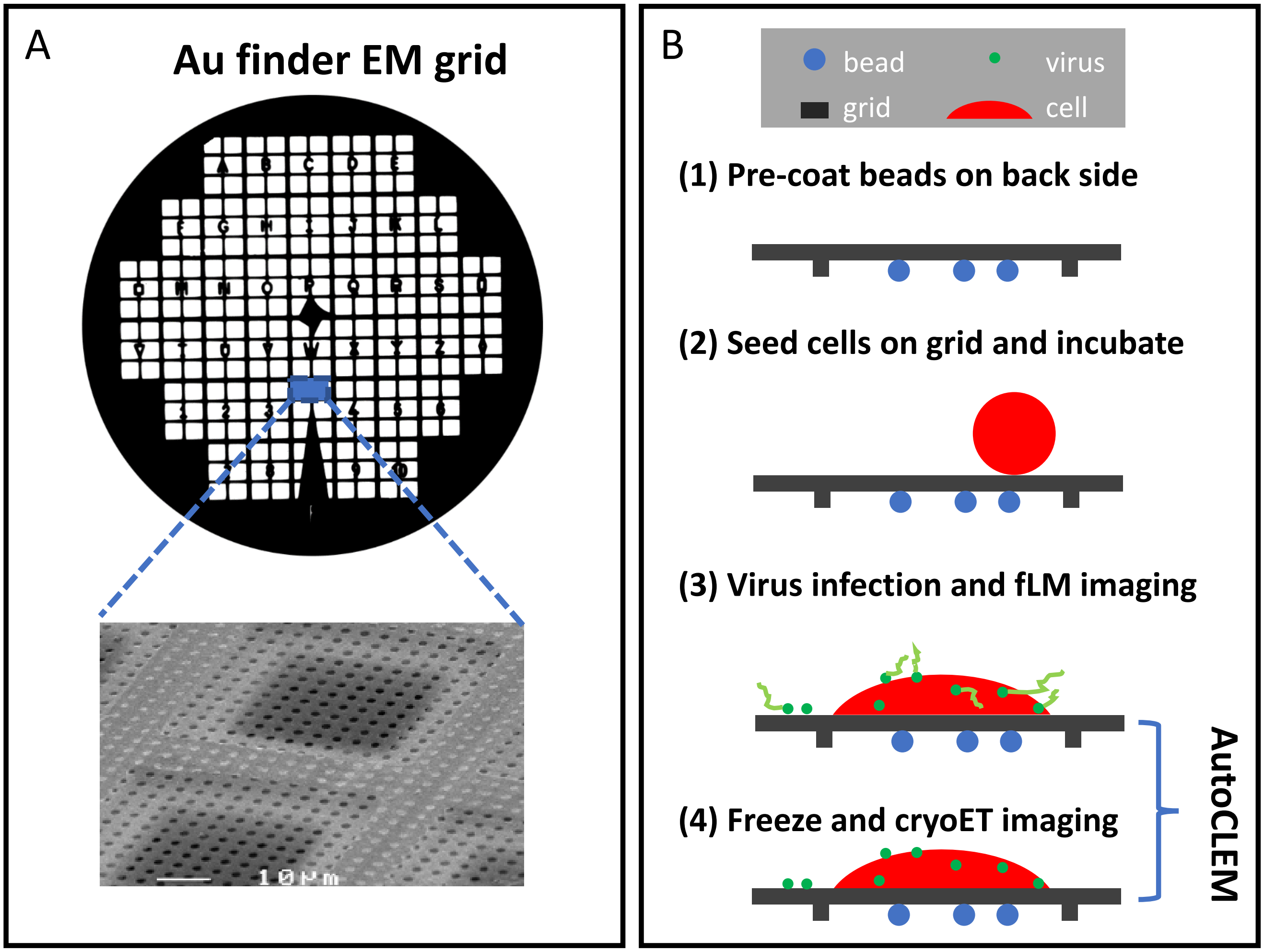

Figure S1| The setup of EM grids and viral infection for AutoCLEM. (A) An Au finder R2/2 Quantifoil grid with 2μm holes. (B) Grid preparation for AutoCLEM. (1) The blue beads are pre-coated on the back side Au finder EM grid. (2) Cells (red) are seeded on the front side and incubated until they are most spreading. (3) Viruses (green) are added and time-lapse live-cell confocal fLM image are recorded. (4) Grids are immediately plunge-frozen for cryoEM correlation and cryoET data collection.

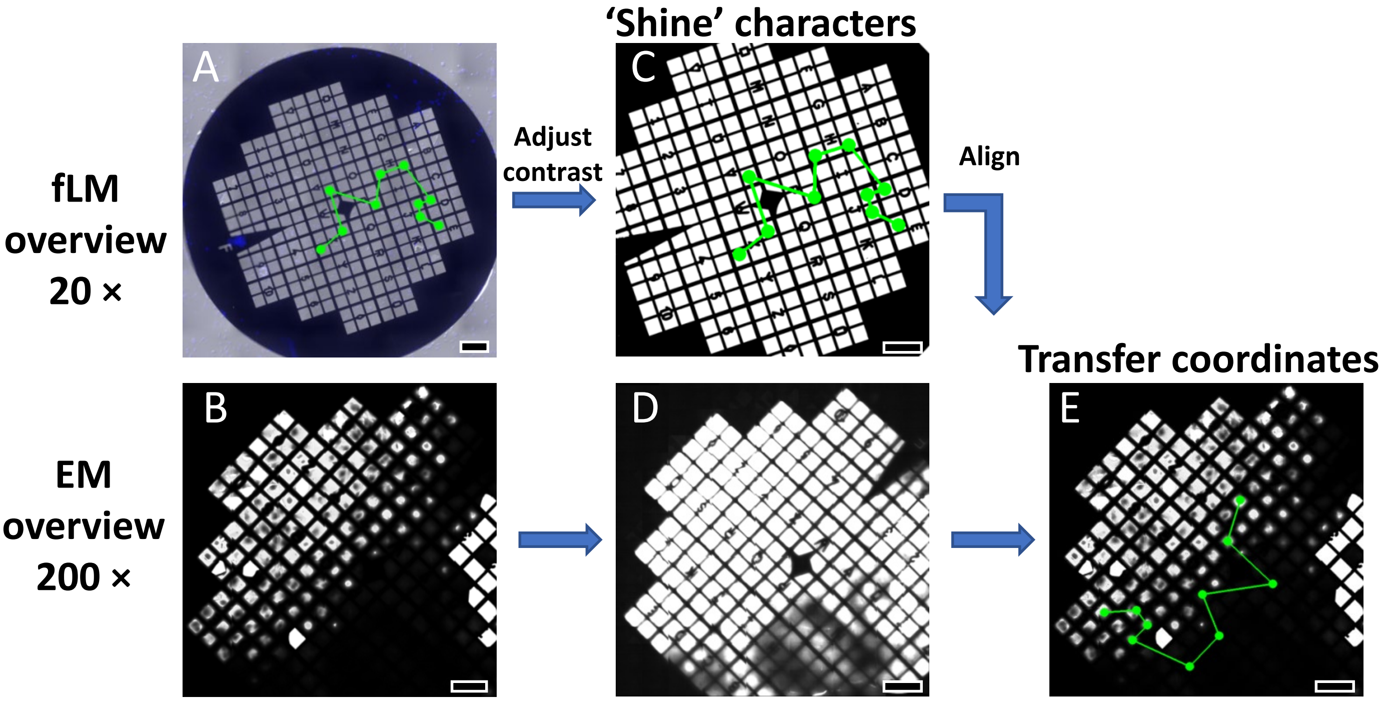

Figure S2| Automatic grid square correlation between fLM and cryoEM. (A) An EM grid was automatically scanned for a 5x5 array with fLM at 20x in DIC mode. The green dots indicate those target squares imaged at fLM. (B) The corresponding overview map recorded at low magnification in cryoEM. (C & D) Processed with thresholding and rescaling. These two images are aligned by using the dominating contrast from grid bar and 'labels'. (E) The squares imaged at fLM (green dots) are automatically mapped onto cryoEM overview map. The scale bars are 20 μm in A-E.

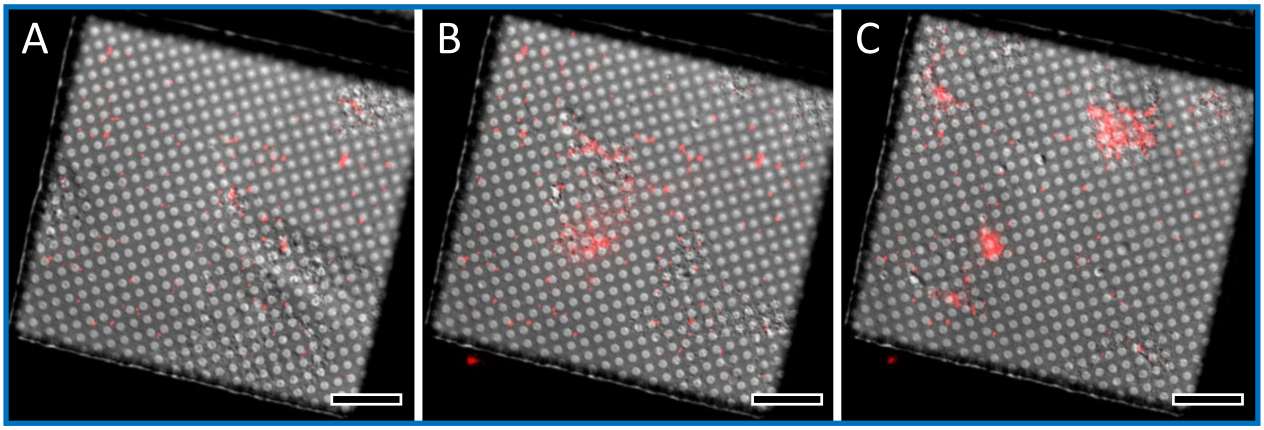

Figure S3| Characterization of fluorescence beads as fiducial markers. (A-C) Overlaid DIC and fLM images of 200 nm beads (shown in red) recorded at 1, 13 and 25 hours after addition of beads to the sample side. The scale bar is 20 μm in A-C.

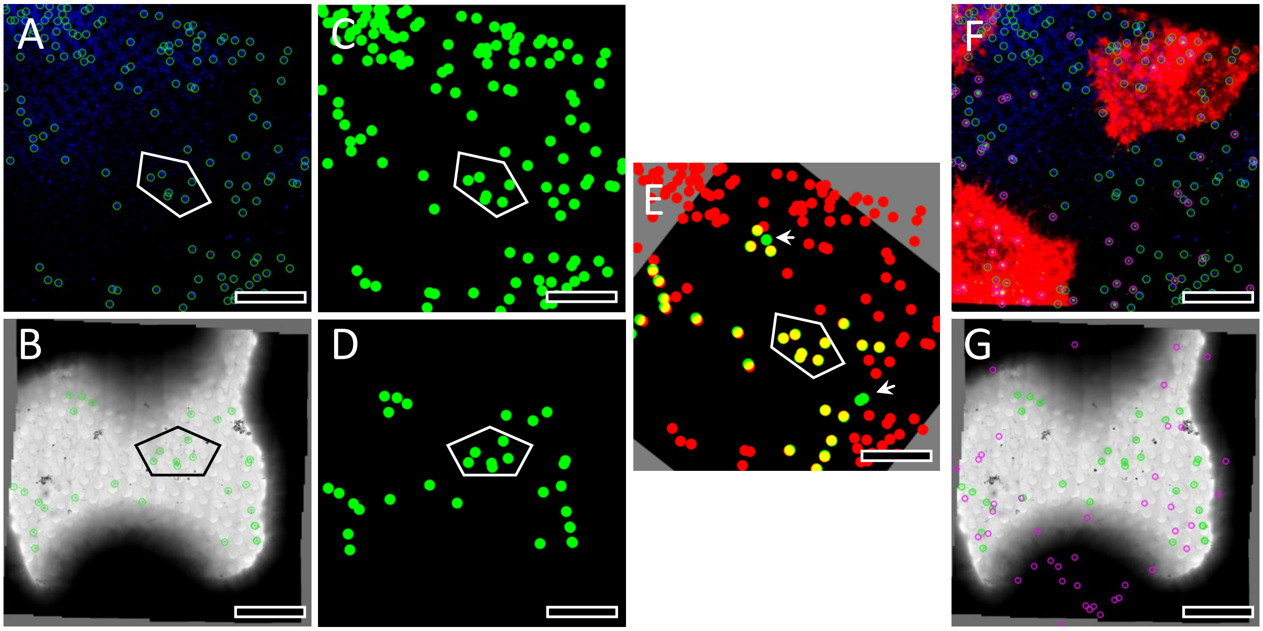

Figure S4| Automatic fluorescent bead picking, correlation with cryoEM and viral particle localization. (A-B) Bead auto-picking from the projection of last confocal fLM image using EMAN2 (A), and from cryoEM grid-square image using IMOD (B). (C-D) Model images generated in IMOD with the bead coordinates from A and B, respectively. The transform matrix is calculated by aligning these two bead-model images using Matlab. (E) The overlay of fLM model beads (red) and the transformed cryoEM model beads (green). Two green dots (arrows) are missing in fLM model image, probably due to weak fluorescence signals. Many red dots are missing in the cryoEM model image, corresponding to mostly thick areas. (F) Automatically picked beads (green circles) and virus particles (pink circles) in the overlaid fLM image, with cells shown in red, beads in blue and viral particles in green. (G) localization of corresponding viral particles (pink circles) in cryo grid-square map, computed from the viral particles coordinates in F (pink circles). The scale bars is 20 μm in A-G.

Table S1: Comparison of time for each step between cryo-AutoCLEM and regular cryo-CLEM

Steps	Regular CLEM	Auto CLEM	improvement
Square correlation	1hr	10 mins	√
Live-cell imaging	40 mins	40 mins	-
Virus localization	2hrs/square	10mins/square	√√

Movie1: confocal live-cell imaging and virus tracking. A typical live-cell imaging started right after the HIV-1 virus (green) were added. The duration was 40 mins with 4 mins interval.

Movie2: Cell eating beads . Live-cell imaging shows the cells take in beads (blue) after settled on the EM grid at the same side with beads. After 24 hours most of beads were taken in by cells.

AutoCLEM Script

```
% align cryo and fluo beads with estimateGeometry by using four cryo images
% update Nav file for serialEM
% the input Nav file has to be converted into Windows Excel first.

clear;
close all;

cd('/disk/grid3');

% input
square              = ['S05';'S06';'S07';'S10';'S11';'S12'];
fnFluoBesds_pre       = 'grid3_end_'; % flu beads on confocal camera
fnFluoBesds_post      = '_flipH_beads.mod';
fnFluoVirus_pre       = 'grid3_end_'; % flu virus on confocal camera
fnFluoVirus_post      = '_flipH_virus.mod';
fnNav                 = 'XF287B_beadsOnly.xlsx';

% output
fnNavUpdated          = 'XF287B_updated_all.nav';


% fnFluoBeadsMod        = 'grid3_end_S12_flipH_beads.mod'; % flu beads on confocal camera
% fnFluoVirusMod        = 'grid3_end_S12_flipH_virus.mod'; % flu virus on confocal camera
% fnNav                 = 'XF287B_beadsOnly.xlsx';
% S_n                   = 'S12';    % for example square 12
%

 % load beads coordinates from cryoEM
[num,text,raw] = xlsread(fnNav);
[rows, columns] = size(num);

% ouput old Nav files
fid = fopen(fnNavUpdated,'w');
formatS = '';
for i = 1:1
   for j = 1:2
      if isnan(num(i,j))
         Nav_updated1{j} = char(text(1,j));
         formatS = [formatS,'%s\t'];
      else
         Nav_updated1{j} = num(1,j);
         if  ~mod(Nav_updated1{i,j},1)
            formatS = [formatS,'%d\t'];
         else
            formatS = [formatS,'%.3f\t'];
         end
      end
   end
   fprintf(fid,[formatS,'\n'], Nav_updated1{:});
end

formatS = '';
for i = 1:1
   for j = 1:74
         Nav_updated2{j} = char(text(2,j));
         formatS = [formatS,'%s\t'];
   end
   fprintf(fid,[formatS,'\n'], Nav_updated2{:});
end

for i = 3:rows
   formatS = '';
   for j = 1:74
      if isnan(num(i,j))
         Nav_updated3{j} = char(text(i,j));
         formatS = [formatS,'%s\t'];
      else
```

```
            Nav_updated3{j} = num(i,j);
            if  ~mod(Nav_updated3{j},1)
                formatS = [formatS,'%d\t'];
            else
                formatS = [formatS,'%.3f\t'];
            end
        end
    end
    fprintf(fid,[formatS,'\n'], Nav_updated3{:});
end


for k=1:size(square,1)
    S_n = square(k,:);
    fnFluoBeadsMod  = [fnFluoBesds_pre,S_n,fnFluoBesds_post];
    fnFluoVirusMod  = [fnFluoVirus_pre,S_n,fnFluoVirus_post];
% load flu coordinates for beads and virus
modParticle1 = ImodModel(fnFluoBeadsMod);
Pt1 = round(imodPoints2Index(getPoints(modParticle1,1)));
FluoBeadsMod = Pt1(1:2,:);
numBeads = length(FluoBeadsMod);

modParticle2 = ImodModel(fnFluoVirusMod);
Pt2=round(imodPoints2Index(getPoints(modParticle2,1)));
FluoViursMod = Pt2(1:2,:);
numVirus = length(FluoViursMod);

indB = strmatch([S_n,'B'],text(:,12));
CryoBeadsStage = num(indB,3:5);
indB_1 = indB(1); % the index for the first bead

% transfer ccordinate from fluo to cryo stage
t2 = estimateGeometricTransform(FluoBeadsMod',CryoBeadsStage(:,1:2),'similar');
FluoViursModTrans = FluoViursMod'*t2.T(1:2,1:2) + repmat(t2.T(3,1:2),numVirus,1);


% output calculated virus coordinates
for i = 1:numVirus
    formatS = '';
    for j = 1:74
        if isnan(num(indB_1,j))
            Nav_updated4{j} = char(text(indB_1,j));
            formatS = [formatS,'%s\t'];
        else
            Nav_updated4{j} = num(indB_1,j);
            if  ~mod(Nav_updated4{j},1)
                formatS = [formatS,'%d\t'];
            else
                formatS = [formatS,'%.3f\t'];
            end
        end
    end
    Nav_updated4{1} = i;                          % label index
    Nav_updated4{2} = 1;                          % color
    Nav_updated4{3} = FluoViursModTrans(i,1);      % coordinates
    Nav_updated4{4} = FluoViursModTrans(i,2);
    Nav_updated4{12} = [S_n,'V',num2str(i)];       % S12V01
    fprintf(fid,[formatS,'\n'], Nav_updated4{:});
end
end

fclose(fid);
```
